# Neutrophil-to-Lymphocyte Ratio, Platelet-to-Lymphocyte Ratio, and Routine Complete Blood Count Components in HELLP Syndrome: A Matched Case Control Study

**DOI:** 10.3390/medicina55050123

**Published:** 2019-05-08

**Authors:** Giovanni Sisti, Andrea Faraci, Jessica Silva, Ruchi Upadhyay

**Affiliations:** Lincoln Medical and Mental Health Center, Department of Obstetrics and Gynecology, 234 E 149th St, Bronx, NY 10451, USA; andrea.faraci@nychhc.org (A.F.); silvaj6@nychhc.org (J.S.); ruchi.upadhyay@nychhc.org (R.U.)

**Keywords:** HELLP syndrome, NLR, PLR, inflammation

## Abstract

*Background and objective*: Neutrophil-to-lymphocyte ratio (NLR) and platelet-to-lymphocyte ratio (PLR) are new readily available inflammatory markers that have been analyzed in pregnancy-induced hypertensive disorders such as preeclampsia. Studies on the NLR/PLR ratio in hemolysis, elevated liver enzymes, low-platelet count (HELLP) syndrome are limited in the current literature. We compared NLR/PLR and other complete blood count (CBC) components between women with HELLP syndrome and women with healthy pregnancies. *Methods*: We conducted a retrospective matched case–control study at a tertiary care hospital in NY (USA) in the time frame between January 2016 and December 2018. The study compared pregnant women with HELLP syndrome (cases) to women with healthy pregnancies in the third trimester (controls), matched by age, body mass index (BMI), parity, and race. Patient with preeclampsia, infection, and fever were excluded. Venous blood samples were obtained as part of the routine work-up at admission for delivery, which included a CBC. The main outcomes were NLR and PLR. The secondary outcomes were hemoglobin, red cell distribution width (RDW), platelet count, mean platelet volume (MPV), neutrophils, lymphocytes. *Results*: There were 14 patients in each group. They were matched by age, race, BMI, and parity. NLR (5.8 vs. 3.6, *p*-value = 0.002) and neutrophil count (10.7 vs. 6.8, *p*-value = 0.001) were higher in women with HELLP compared to controls. PLR (34 vs. 130.2, *p*-value < 0.001) and platelet count (71 vs. 223, *p*-value < 0.001) were lower in the study group compared to controls. *Conclusions*: NLR was higher, and PLR was lower in women with HELLP syndrome. These inflammatory markers can be incorporated into the diagnostic algorithm for HELLP syndrome. Future studies are needed to evaluate their ability to predict HELLP syndrome.

## 1. Introduction

Hemolysis, elevated liver enzymes, and low-platelet count (HELLP) syndrome was first described in the seminal report by Weinstein in 1982 as the occurrence of hemolysis, elevated liver functions, and low platelet count in the third trimester of pregnancy [1]. It occurs in up to 0.9% of all pregnancies in the third trimester or in the immediate postpartum period and it is associated with adverse maternal and neonatal outcomes [2,3,4].

The exact pathological mechanisms involved in the development of HELLP syndrome have never been fully demonstrated: we know that there is a diffuse endothelial cell damage and a limited vessel involvement, especially in the liver, that lead to hemolysis, red cell fragmentation, schistocytes, and Burr cells. In addition, the activated platelets adhere to damaged vascular endothelial cells, with subsequent platelet consumption [5].

There is still a debate on whether HELLP must be considered a severe form of preeclampsia or a separate disease entity [6,7].

Laboratory investigations and the clinical picture of preeclampsia and HELLP syndrome are different. In HELLP syndrome, the inflammatory reaction is more severe and attacks preferentially the liver and the coagulation system. Hypertension is absent in 10–20% of HELLP patients, whereas it is always present in patients with preeclampsia [6,7].

Preeclampsia appears to be associated with a higher extent of inflammation than uncomplicated pregnancy [8]; preeclamptic patients have an increase in leukocyte count, mainly neutrophils, and a decrease in lymphocytes [8,9].

Different studies showed that thrombocytes are significantly decreased, and mean platelet volume is significantly increased in women with preeclampsia [9,10,11].

An increase in neutrophils and a decrease in lymphocytes resulted in an increased neutrophil-to-lymphocyte ratio (NLR) in preeclampsia [11,12] in most, but not all, available studies [13]. The lower number of lymphocytes and the higher number of platelets led to an increased platelet-to-lymphocyte ratio (PLR) in some studies, whereas, in other studies, the PLR did not differ between cases and controls [13,14,15].

The role and contribution of inflammation towards neutrophilic activation and endothelial dysfunction during the development of HELLP syndrome have, however, been largely overlooked.

To date, the levels of NLR and PLR in HELLP syndrome have not been reported.

Considering the alterations in the peripheral levels of NRL and PLR in previous studies on preeclampsia, we hypothesized that NRL and PLR levels were also altered in women with HELLP syndrome.

We compared NLR and PLR levels in women with HELLP syndrome to those in women with healthy pregnancies in the third trimester. We also investigated the levels of other complete blood cell count (CBC) components in both groups.

## 2. Material and Methods

We conducted a retrospective matched case–control study at a tertiary care hospital in NY (USA) in the time frame between January 2016 and December 2018. The institutional review board (IRB) approved the study (IRB # 19-005 on 2 January 2019). The study compared pregnant women with HELLP syndrome (cases) to healthy pregnant patients (controls). The inclusion criterion was being in the third trimester of pregnancy. The two groups were matched by age, body mass index (BMI), parity, and race. HELLP syndrome was defined according to the Tennessee Classification System diagnostic criteria: LDH > 600 U/L, AST ≥ 70 U/L, and platelets < 100 × 10(9)/L [16].

We included as cases also patients with partial HELLP syndrome, who required two out of three criteria [17,18].

Patients with preeclampsia, infection, and fever were excluded. The venous blood samples were obtained as part of the routine work-up at admission for delivery, which includes a CBC. Demographics and clinical information were recorded and included age, BMI, gravidity, parity, race, birthweight, gestational age at delivery, gestational age at CBC collection, mode of delivery. Gestational age was noted as number of weeks, followed by number of days of the week—weeks.days.

The main outcomes were NLR and PLR. The secondary outcomes were hemoglobin, red cell distribution width (RDW), platelet count, mean platelet volume (MPV), neutrophils, lymphocytes. CBC values were analyzed with the hematology analyzer Beckman Coulter.

Statistical analysis was performed with SPSS v. 22.0 (IBM, Chicago, US). Continuous variables were expressed with median (25°–75° percentile); categorical variables were indicated by percentage.

We assessed the normality of data with the Shapiro–Wilk test: for all variables, the test had a *p*-value below 0.05, indicating that all data significantly deviated from a normal distribution.

To compare the demographics and outcomes variables between the cases and the controls, we used the non-parametric Mann–Whitney test.

A *p*-value of less than 0.05 was considered statistically significant.

## 3. Results

There were 28 patients in total. Median age was 28 years old (24–31); median BMI was 30 kg/m^2^ (26–32), 20% were Afro-American, and 80% Hispanic; 57% were multipara, median birthweight was 3085 gr (2512–3597 gr), median gestational age at delivery was 38 years (35–39). No patients had a history of chronic hypertension or preeclampsia.

Each group had 14 patients, matched by age, race, BMI, and parity (Table 1).

Three patients had partial HELLP, and 11 patients had complete HELLP syndrome.

The gestational age at delivery was lower in the HELLP group [35.0 (29.0–37.0) vs. 39.0 (38.1–40.0) weeks.days, *p* = 0.0001].

Birthweight was lower in the HELLP group [2450 (1030–2665) vs. 3572 (333.7–3700)] gr, *p* < 0.0001).

The gestational age at CBC collection was lower in the HELLP group [34.0 (28.2–37.1) vs. 39.0 (38.0–40.0) weeks.days, *p* < 0.001].

NLR(5.8 vs. 3.6, *p*-value = 0.002) and neutrophil count (10.7 vs. 6.8, *p*-value = 0.001) were higher in women with HELLP syndrome compared with the control group (Table 2). PLR (34 vs. 130.2, *p*-value < 0.001) and platelet count (71 vs. 223 10^9^/lt, *p*-value < 0.001) were lower in the study group compared with the control group (Table 2).

## 4. Discussion

Our results indicate that NLR was increased and PLR was decreased in women with HELLP syndrome.

In addition, neutrophil count was higher, while platelet count was lower in patients with HELLP syndrome.

The modifications in NLR and PLR have been extensively studied in hypertensive disorders of pregnancy, but ours is the first study comparing NRL and PLR between patients with HELLP syndrome (excluding mere preeclampsia) and women with healthy third-trimester pregnancies.

The association between NLR/PLR and preeclampsia produced mixed results in previous studies [11,12,13].

Gogoi et al., in 2017, compared 67 women with preeclampsia to 67 controls and found higher NLR and PLR in the cases (6.8 vs. 3, *p* = 0001 and 14.18 vs. 9.54, *p* = 0.012) [14].

Mannerts et al., in 2019, compared 169 patients with preeclampsia or HELLP syndrome to 1886 controls and found higher NLR (6.79 vs. 3.6 *p* < 0.001) and lower PLR (91.47 vs. 129.05, *p* = 0.0003) in the preeclamptic group compared to the controls [11].

Yucel et al., in 2016, showed non-significant differences regarding NLR between control subjects and patients with mild preeclampsia, control subjects and patients with severe preeclampsia, and patients with mild preeclampsia and those with severe preeclampsia; they found lower PLR in patients with severe preeclampsia compared to controls (89.05 vs. 102.2, *p* = 0.007), but not between controls and patients with mild preeclampsia or between patients with mild or severe preeclampsia [15].

Cintesun et al., in 2018, did not find any statistically significant difference regarding NLR and PLR between 64 patients with preeclampsia and 66 women with healthy pregnancies in the third trimester [13].

We speculate that HELLP syndrome and severe preeclampsia share the same hyper-inflammatory activation that leads to higher NLR and lower PLR, compared to controls. 

Preeclampsia develops with a defective placentation, with excessive innate/adaptive immune activation and inflammation at the maternal–fetal interface.

In preeclamptic patients, there is a shift from Th2 to Th1 lymphocytes, with decreased immune tolerance [8,9,10,11,12,13,14,15,16,17,18,19].

In HELLP syndrome, a stronger and more specific activation of the immune system has been hypothesized: in HELLP syndrome the coagulation system is directly under attack, and the platelet count reaches much lower levels than in preeclampsia [13,14,15].

Leukocytosis is a common finding accompanying HELLP syndrome, consequential to the inflammatory attack to the liver. The higher levels of neutrophils and lower levels of platelets found in patients with HELLP syndrome confirm previous studies on pregnancy hypertensive disorders and other obstetrical pathologies [20].

A strength of our study is to have distinguished HELLP syndrome from preeclampsia: we compared HELLP patients to controls, excluding any patient with mere preeclampsia. We were able to highlight the unique features of this syndrome.

Limitations of this study are the lack of a comparison group with preeclampsia and the low power of the study, given the low number of participants. In addition, we did not perform a sub analysis for the partial HELLP syndrome vs. the complete one because of the low number of patients with partial HELLP syndrome (3 out of 14).

There are similarities between HELLP syndrome and other pregnancy hypertensive disorders and obstetrical inflammatory pathologies [20].

However, the exact mechanisms of the inflammatory process in the development of HELLP syndrome remain unclear.

## 5. Conclusions

The present study found that the inflammatory marker NLR was higher and the PLR was lower in women with HELLP syndrome. These markers could be incorporated into the diagnostic algorithm for HELLP syndrome: they could be a useful addition to the standard diagnostic criteria currently used, if confirmed by larger studies.

Future studies are needed to evaluate their ability to predict HELLP syndrome.

## Figures and Tables

**Table 1:** Demographic/matching variables values in cases and controls. * Mann–Whitney test. ** Chi-square test. GA: gestational age (noted as number of weeks, followed by number of days of the week, i.e., weeks.days), HELLP: hemolysis, elevated liver enzymes, and low-platelet count syndrome, BMI: body mass index.

	HELLP	Controls	*p*-Value
Age (years)	26.3 [22.3–30.3]	29.2 [22–34.3]	NS *
BMI (kg/m^2^)	31.1 [25.1–35.1]	28.5 [27.1–32.1]	NS *
Parity >1	6/14	10/14	NS **
Afro-American/Hispanic	3/14	3/14	NS **
GA at delivery (weeks.days)	35.0 [29.0–37.0]	39.0 [38.1–40.0]	0.001 *
Neonatal birthweight (gr)	2450 [1030–2665]	3572 [3333.7–3700]	<0.001 *

**Table 2:** Routine complete blood count (CBC), neutrophil-to-lymphocyte ratio (NLR), and platelet-to-lymphocyte ratio (PLR) values in cases and controls. RDW: red cell distribution width, MPV: mean platelet volume.

	HELLP	Controls	*p*-Value (Mann-Whitney Test)
GA at CBC collection (weeks.days)	34.0 [28.2–37.1]	39.0 [38.0–40.0]	<0.001
NLR	5.8 [3.7–16]	3.6 [2.7–6]	0.02
PLR	34 [24.4–81.2]	130.2 [115.5–146.5]	<0.001
Hemoglobin (gr/dl)	11.1 [10.7–12.2]	11.8 [11.6–12.3]	NS
Platelet (10^9^/lt)	71.5 [53.7–79.5]	223.5 [179–245]	<0.001
RDW (%)	11.1 [10.7–12.2]	11.8 [11.6–12.3]	NS
MPV (fL)	9.1 [7.8–9.7]	9.4 [7.8–9.9]	NS
Neutrophil (absolute count)	10.7 [9–13]	6.8 [5–8.8]	0.001
Lymphocyte (absolute count)	1.7 [1–2.5]	1.6 [1.2–1.8]	NS
